# A collection of cannabinoid-related negative findings from autaptic hippocampal neurons

**DOI:** 10.1038/s41598-023-36710-3

**Published:** 2023-06-13

**Authors:** Alex Straiker, Michaela Dvorakova, Taryn Bosquez-Berger, Jaroslav Blahos, Ken Mackie

**Affiliations:** 1 Department of Psychological and Brain Sciences, Gill Center for Biomolecular Science, Program in Neuroscience, Indiana University, Bloomington, IN 47405 USA; 2 Department of Molecular Pharmacology, Institute of Molecular Genetics of the Czech Academy of Sciences, Videnska 1083, 142 20 Prague 4, Czech Republic

**Keywords:** Drug discovery, Molecular biology, Neuroscience

## Abstract

Autaptic hippocampal neurons are an architecturally simple model of neurotransmission that express several forms of cannabinoid signaling. Over the past twenty years this model has proven valuable for studies ranging from enzymatic control of endocannabinoid production and breakdown, to CB_1_ receptor structure/function, to CB_2_ signaling, understanding ‘spice’ (synthetic cannabinoid) pharmacology, and more. However, while studying cannabinoid signaling in these neurons, we have occasionally encountered what one might call ‘interesting negatives’, valid and informative findings in the context of our experimental design that, given the nature of scientific publishing, may not otherwise find their way into the scientific literature. In autaptic hippocampal neurons we have found that: (1) The fatty acid binding protein (FABP) blocker SBFI-26 does not alter CB1-mediated neuroplasticity. (2) 1-AG signals poorly relative to 2-AG in autaptic neurons. (3) Indomethacin is not a CB1 PAM in autaptic neurons. (4) The CB1-associated protein SGIP1a is not necessary for CB1 desensitization. We are presenting these negative or perplexing findings in the hope that they will prove beneficial to other laboratories and elicit fruitful discussions regarding their relevance and significance.

## Introduction

Autaptic hippocampal neurons are an architecturally simple model of neurotransmission. Autaptics, as we shall refer to them, also possess all of the machinery required for several forms of retrograde cannabinoid signaling including depolarization-induced suppression of excitation (DSE)^1^. Over the course of 20 years, we have used this model system to study the components of the cannabinoid signaling system in detail. Experiments using these cultures have ranged from investigating enzymatic control of endocannabinoid production and breakdown^2,3^, to CB_1_ mutants, species-differences, and splice variants^4^, to CB_2_ signaling^5^. More recently we have made extensive use of this model system to study cannabinoid pharmacology including ‘spice’ pharmacology^6,7^ and allosteric modulators of the CB1 receptor^8,9^. In total we have published more than 25 cannabinoid-related papers using the autaptic model. It is the nature of scientific research to preferentially publish positive, rather than negative, results. However, in the course of studying cannabinoid signaling in these neurons we have occasionally encountered what one might call ‘interesting negatives’ or orphan datasets that may not amount to publishable units on their own. Here we have compiled a series of such notable findings relating to transport and isomerization of endocannabinoids, allosteric modulation and desensitization of the cannabinoid CB1 receptor.

### 2-AG transport by fatty acid binding proteins (FABPs)

One of the continuing mysteries of cannabinoid signaling has to do with how endogenous cannabinoids make their way from the inside postsynaptic dendrites to the presynaptic terminal to activate the membrane bound CB1 receptors. Synthesis of 2-AG, the endocannabinoid that is clearly implicated in autaptic cannabinoid signaling, is considered to occur via the activity of diacylglycerol lipases^3^. What happens next is largely a mystery, but chaperones and transporters have been proposed at various instances. The rationale is that as lipophilic messengers, the intracellularly synthesized endocannabinoids would be reluctant to cross the membrane and that a transporter or chaperone might facilitate or even guide endocannabinoids accordingly. The compounds VDM11 and the paracetamol metabolite AM404 were proposed early on as anandamide transport inhibitors^10,11^. The hypothesized action of AM404 on anandamide transport is still considered a potential mechanistic basis for the analgesic effects of acetaminophen^12^. A family of binding proteins have also been proposed to act as chaperones for anandamide to facilitate their transfer to the fatty acid amidohydrolase (FAAH) that resides in the smooth endoplasmic reticulum^13^. These fatty acid binding proteins (FABPs) have been implicated in several important physiological functions (reviewed in^14^). The autaptic model is an ideal testbed to evaluate a compound affecting endocannabinoid transport to determine the role of endocannabinoid transport in DSE.

Once 2-AG is in the presynaptic membrane it rapidly engages the CB1 receptor. We have shown that the characteristic decay of DSE in autaptics is determined by the complement of metabolizing enzymes, in this case presynaptic monoacylglycerol lipase (MAGL^2,15^), suggesting that uptake of 2-AG is not rate-limiting in its elimination, rather 2-AG hydrolysis determines the speed of recovery from DSE. A provocative hypothesis has been proposed for 2-AG trafficking. As noted above, the question of how 2-AG crosses the synapse has not been experimentally addressed. If FABPs act as transporters intracellularly, might they also act as part of a trans-synaptic ferry system? FABP5 in particular has recently been implicated in both 2-AG and anandamide homeostasis in the striatum^16^ and dorsal raphe nucleus^17^. Important for this study, FABP mRNA expression has been reported in hippocampal pyramidal cells^18^. Block of 2-AG transport should have clear consequences for DSE in autaptic neurons.

### The functional role of 1-AG in a neuronal model

One of the first publications to describe a functional role for 2-AG in cannabinoid signaling^19^ devoted some attention to a curious isomerization event that they observed: 2-AG spontaneously and substantially isomerized to 1-AG, also referred to as 1(3)-AG. Stella et al.^19^ estimated that the 1-AG that they detected could be entirely accounted for by non-enzymatic isomerization of 2-AG, since they found that 43% of their 2-AG standard isomerized under the same experimental conditions. They therefore made a correction to the 2-AG effects based on this control isomerization. In addition, they also tested whether 1-AG had activity. Stella et al.^19^ found 1-AG to be at least as efficacious as (and perhaps more potent than) 2-AG in inhibiting forskolin-stimulated production of cAMP. Moreover, they also found 1-AG to be capable of inducing long-term potentiation in brain slices.

### Indomethacin as a positive allosteric modulator at CB1

Indomethacin is a widely used non-steroidal anti-inflammatory drug that can be used for pain and fever relief. First described in 1963^20^, indomethacin is thought to act via inhibition of both cyclooxygenase 1 (COX1) and COX2, inhibiting the production of prostaglandins (reviewed in^21^). It has been reported that indomethacin acts as a positive allosteric modulator at cannabinoid CB1 receptors^22^ to enhance cannabinoid signaling, though the most pronounced allosterism occurred with synthetic rather than endogenous cannabinoids. We have published several studies of allosteric modulator function in the autaptic model, including putative negative (NAM) and positive (PAM) allosteric modulators^8,9,23^. We tested whether indomethacin acted as a PAM in autaptic neurons, focusing on 2-AG signaling.

### SH3 domain GRB2 like endophilin interacting protein 1 (SGIP1) and desensitization in a neuronal model

Several proteins have been reported to interact with CB1 to modify its functions. Some of these are classical proteins such as arrestins that have a broad role in regulating GPCR function, but some have been proposed to have specific interaction with CB1 receptors. The earliest of these was cannabinoid receptor interacting protein 1 (CRIP1)^29^, but more recently, SH3 domain GRB2 like endophilin interacting protein 1 (SGIP1) has also been proposed to negatively regulate internalization of several cargoes from the cell surface^30^ including CB1^31^. In the case of CB1 SGIP1 binds the CB1 C-terminus and enhances its association with β-arrestin 2 and GRK3^32^ and decreases CB1-mediated ERK1/2 signaling while leaving G-protein signaling unaffected. We tested the effect of SGIP1 deletion on CB1 signaling in autaptic cultures.

## Methods

### Animals

All animal care and experimental procedures used in this study were approved by the Institutional Animal Care and Use Committee of Indiana University and conform to the Guidelines of the National Institutes of Health on the Care and Use of Laboratory Animals. Experiments complied with ARRIVE guidelines. For neuronal cultures described below, hippocampal neurons were obtained from mice (at postnatal day 0–2, killed via rapid decapitation without anesthesia) as described previously^33^.

### Hippocampal culture preparation

All procedures used in this study were in accordance with applicable local laws and European Union regulations, approved by the Institutional Animal Care and Use Committee of Indiana University and conform to the Guidelines of the National Institutes of Health on the Care and Use of Animals. Autaptic hippocampal neurons were cultured as described previously^34,35^. Neurons were isolated from CA1-CA3 regions of mouse hippocampi (postnatal day 0–2) and plated on a previously prepared feeder layer of astrocytes^33^. Neuronal cultures were kept in high glucose (20 mM) DMEM containing 10% horse serum and used for recording after 8 days in culture. Neurons were used only up to 14 days after isolation.

### Electrophysiology

All experiments were performed on isolated autaptic neurons. The cells were kept at room temperature for the whole time of the recording and were used within three hours after removal from their culture media. Whole cell patch-clamp recordings were performed using a HEKA Triple Patch Clamp EPC10 amplifier (HEKA Elektronik, Lambrecht/Pfalz, Germany) and recording electrodes were filled with intracellular solution (121.5 mM KGluconate, 17.5 mM KCl, 9 mM NaCl, 1 mM MgCl_2_, 10 mM HEPES, 0.2 mM EGTA, 2 mM MgATP, and 0.5 mM LiGTP). The extracellular solution contained 119 mM NaCl, 5 mM KCl, 2.5 mM CaCl_2_, 1.5 mM MgCl_2_, 30 mM glucose, and 20 mM HEPES. The flowrate of the solution through the chamber was ~ 3 ml/min.

2-AG (Cayman Chemical, Ann Arbor, MI, USA) stock was kept at -80 °C and diluted at the final concentration into extracellular solution at the day of experiment. DSE was induced after establishing of a 10–20 s 0.5 Hz baseline. For DSE dose–response experiments depolarization to 0 mV for 50 ms, 100 ms, 300 ms, 500 ms, 1 s, 3 s, 10 s. Each step was followed by resumption of a 0.5 Hz stimulus protocol for 20–80 + seconds to allow the excitatory postsynaptic currents (EPSCs) to recover to baseline. Values prior to depolarization were normalized to 1 and the suppression of excitation values are presented as fractions of 1.

For 2-AG concentration–response experiments the membrane potential was held at -70 mV and EPSCs were triggered every 20 s with a 1 ms depolarizing step. After establishing a 5 min baseline without drug, 2-AG was added to the cells in subsequently higher concentrations (1 nM, 10 nM, 100 nM, 1 µM, 5 µM) and the evoked EPSC was continuously recorded. The flow rate of the solution through the chamber was ~ 3 ml/min and the cells were treated at each drug concentration for 5 min. Relative EPSC charge data are presented as proportions relative to baseline. For desensitization experiments neurons were incubated in 100 nM WIN55,212–2 (WIN, 0.001% DMSO) overnight. After the overnight treatment cells were washed for at least 20 min before they were used for recording of the DSE dose–response (as described above).

### Flamindo cAMP assay

#### Cell culture and transfection

CHO-K1 cells were cultured in high glucose Dulbecco’s Modified Eagle Medium/Nutrient Mixture F12 (Ham’s Medium) (Thermo Fisher Scientific, Waltham, MA, USA), supplemented with 10% fetal bovine serum and a 1% Pen/Strep solution. Cultures were maintained at 37 °C with an atmosphere of 5% CO_2_. For the imaging experiments, the cells were dissociated using trypsin–EDTA (0.05%) and cultured on poly-D-lysine pre-coated 18 mm glass coverslips in 12-well plates. One day post-plating, the cells were transfected with the rat CB1 (rCB1) receptor, the fluorescent protein EYFP, and the red fluorescent cAMP indicator, Pink Flamindo, using Lipofectamine 2000 Transfection Reagent (Thermo Fisher Scientific)^36^. After 3.5 h, the transfection reagent was replaced with cell culture media and the cells were used for experiments within two days of transfection.

#### Cell imaging and cAMP binding assay

CB1-transfected CHO-K1 cells, were imaged in an extracellular solution containing (mM) NaCl 119, KCl 5, CaCl_2_ 2, MgCl_2_ 1, glucose 30 and HEPES 20, pH 7.4, using a Nikon inverted microscope fitted with a Hamamatsu Flash 4 camera and Nikon Elements AR acquisition software. Drugs were initially prepared as a stock in DMSO or 100% ethanol, then diluted using extracellular solution to their final concentration and used on the same day. For experiments using the transfected CHO-K1 cells, a drug concentration of the CB1 agonist CP55,940 (1 nM–1 µM) was individually applied or co-applied with indomethacin (1 µM), followed several minutes later by the adenylyl cyclase activator, forskolin (Fsk; 100 µM).

Images were acquired every 30 s for 15 min and then analyzed using FIJI software (https://imagej.net/software/fiji/downloads) with the 1-click ROI manager plugin^37^, to measure changes in fluorescence intensity. Target cells were chosen by selecting the first image in the series, increasing the brightness, and marking cells that exhibited a baseline Pink Flamindo fluorescence. An occasional population of (< 5%) cells exhibited a very high baseline fluorescence relative to the general transfected cell population. These cells were excluded from analysis since they were close to saturation. The mask of identified cells (typically 15–25 per experiment) was then be applied to the image series. Baseline fluorescence intensity was normalized to 100 based on the first two minutes of the time series. Each plotted point on a time series represents the average fluorescent intensity (AFI) + /- SEM of 3 independent experiments, at a specific time point. Statistically significant differences in these responses were taken as non-overlapping 95% confidence intervals. For a given experimental treatment, a same-day Fsk-only experimental control was included, and the experimental results were compared to their respective same-day controls for reference. Calculation of the EC_50_ for CP55,940.alone, and in combination with indomethacin, were done using GraphPad Prism 9.

#### Drugs

SBFI26 was kindly provided by Dr. Martin Kaczocha (Stony Brook University, Stony Brook, NY). 2-AG was purchased from Cayman Chemical (Ann Arbor, MI). WIN55212-2 was purchased from Sigma Aldrich (St. Louis, MO).

## Results

### The FABP blocker SBFI-26 does not alter cannabinoid signaling in autaptic hippocampal neurons

**Figure 1:**
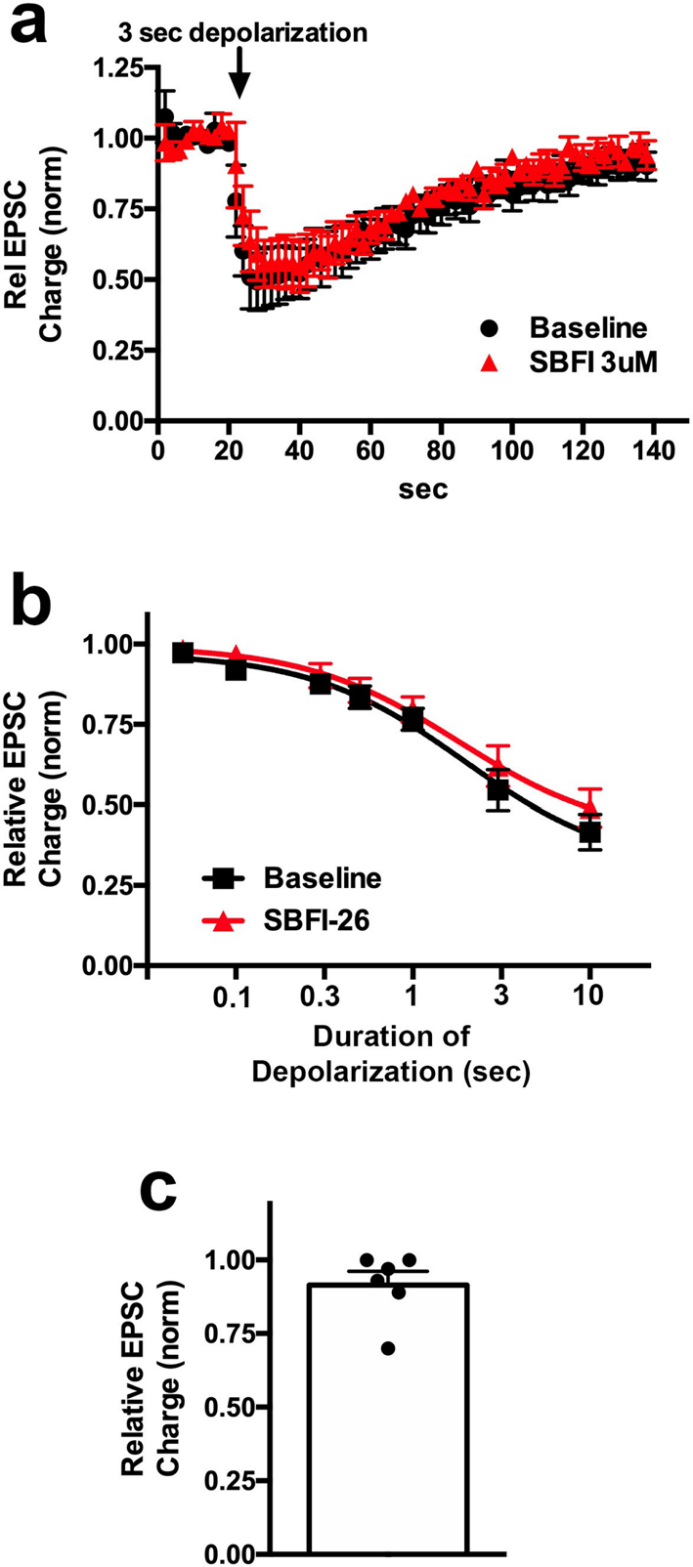
The FABP5/7 inhibitor SBFI26 does not alter DSE responses in autaptic hippocampal neurons. (**a**) Averaged DSE responses show the time-courses in response to a 3 s depolarization before (black) and 5 min after treatment with SBFI26 (3 µM, red). (**b**) Depolarization-response curve shows DSE responses are unaltered by SBFI. (**c**) SBFI26 had no direct effect on EPSC amplitude.

As noted above, FABPs have been proposed to play a role in 2-AG trafficking in neurons. We tested SBFI-26, a compound with antinociceptive properties that blocks FABP5 and FABP7^38^ by determining if it affects the amount of depolarization necessary to elicit DSE, quantified by the ED50, the duration of depolarization necessary to elicit 50% of maximal DSE and the maximal DSE. SBFI-26 did not alter DSE responses (Fig. 1a,b; ED50 baseline duration of depolarization (s (95% CI)): 1.9 (1.2–3.3); After SBFI-26 (3 µM): 1.8 (1.4–2.4)). SBFI-26 also had no direct effect on EPSC amplitude (Fig. 1c; Direct effect of SBFI-26 on EPSC size 3 µM, 5 min; relative to baseline (1.0 = no effect): 0.92 ± 0.05 n = 6; NS by one-sample t-test).

### 1-AG inhibits synaptic transmission in autaptic hippocampal neurons poorly relative to 2-AG

**Figure 2:**
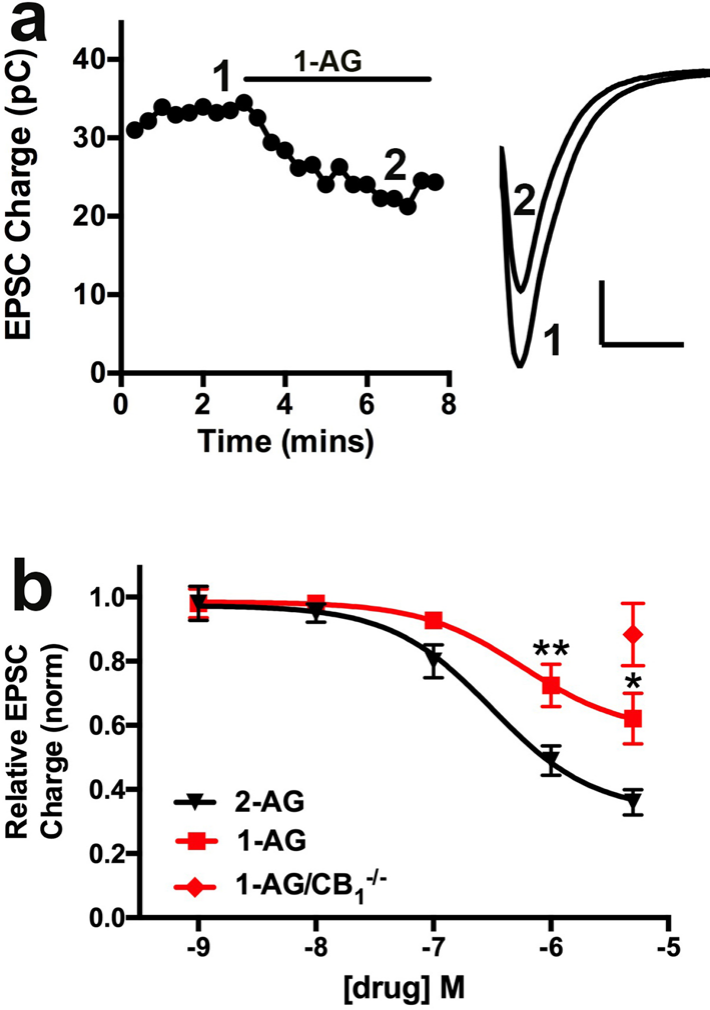
1-AG signals poorly relative to 2-AG in autaptic hippocampal neurons. (**a**) Sample time course showing the effect of 1-AG (1 µM) treatment on evoked EPSCs. Right panel shows corresponding EPSCs before (1) and in the presence of (2) 1-AG. (**b**) Concentration response curves show inhibition of excitatory postsynaptic currents with increasing concentrations of 1-AG (red squares) or 2-AG (black triangles). The curve for 1-AG is shifted to the right by ~ 1 order of magnitude. Red diamond shows the response to 1-AG at 5 µM in CB_1_^−/−^ neurons. **, p < 0.01 2-way ANOVA with Bonferroni post hoc test. Scale bars for inset in A: nA, 50 ms.

Given the centrality of 2-AG in cannabinoid signaling, it is important to determine the disposition of 1-AG. Given the report by Stella et al. that 1-AG represented ~ 40% of the measured acylglycerols^19^, it is important to know how 1-AG signals. There are two main questions regarding 1-AG: 1) Is 1-AG broadly functional and potent as suggested by the results of Stella et al. and, if so, 2) Is 1-AG physiologically relevant? To address these questions, we tested the efficacy of 1-AG to engage CB1 receptors in autaptic hippocampal neurons, recording from neurons to obtain baseline EPSCs, and then treating the cells with 1-AG at various concentrations (Fig. 2a). As shown in Fig. 2a,b, 1-AG inhibits EPSCs in a concentration-dependent manner but appears to be substantially less potent than 2-AG. The EC50 for 1-AG is difficult to calculate accurately because it has not reached a maximal effect at 5 µM, however the inhibition by 1-AG at 1 µM is substantially less than inhibition by 2-AG at the same concentration (p < 0.05 at 1 µM and 5 µM, 2-way ANOVA with Bonferroni post hoc test, n = 8). It should be noted that the commercially supplied 1-AG came as a 9:1 1-AG/2-AG stock, meaning that, if 1-AG was inactive, the application of the commercially supplied 1-AG/2-AG can be expected to elicit an order of magnitude lower 2-AG-dependent signaling. Thus, the inhibition seen with application of 1 µM 1-AG may be due to the presence of 100 nM 2-AG, which produces a comparable inhibition. 1-AG (5 µM) did not significantly inhibit EPSCs in CB_1_^−/−^ neurons, indicating that 1-AG inhibition was CB_1_-dependent (Fig. 2b; relative EPSC charge after 1-AG (± SEM): 0.88 ± 0.10, n = 6, NS by one-sample t-test vs. baseline (1.0)).

### Indomethacin does not act as a positive allosteric modulator (PAM) in autaptic neurons

As noted above, indomethacin has been reported to act as a PAM at CB1 receptors^22^. The simplest test for PAM activity in the autaptic model is to examine whether indomethacin enhances native CB1 responses to exogenously applied 2-AG. We have previously tested a range of 2-AG concentrations and found that 1 µM serves as a concentration that induces a clear but submaximal inhibition of EPSCs.

**Figure 3:**
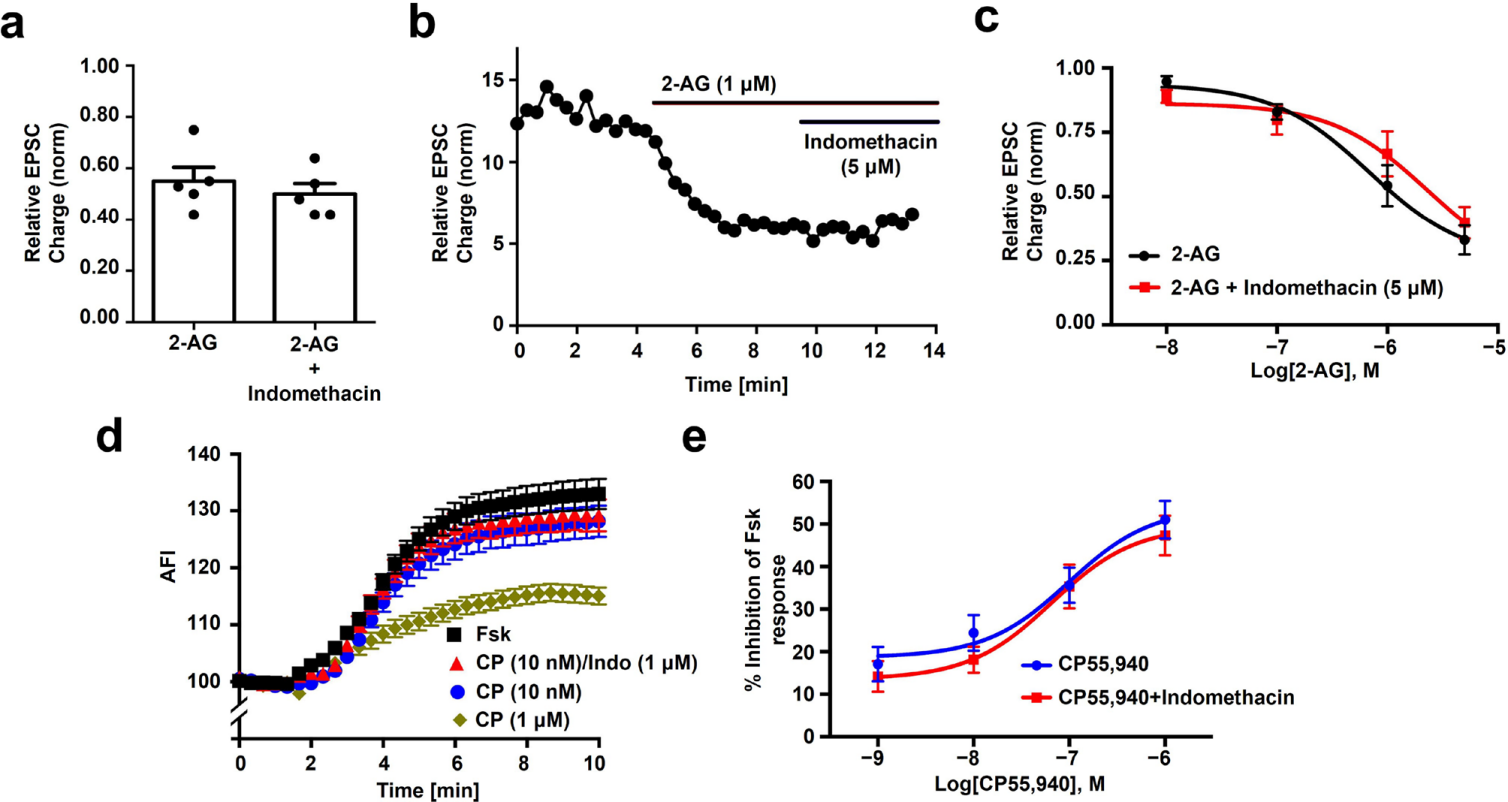
Indomethacin does not act as a positive allosteric modulator in autaptic neurons or in a cAMP assay. (**a**) Bath-applied 2-AG (1 µM) is not enhanced by indomethacin (5 µM). (**b**) Sample time course from a). (**c**) 2-AG concentration response is not altered by 5 min pretreatment with indomethacin (5 µM). (**d**) Sample time courses showing cAMP responses to forskolin under various treatment conditions. (**e**) Summary of cAMP responses showing that CP55,940 responses are not altered by inclusion of indomethacin (1 µM).

We found that 5 µM indomethacin did not enhance the effects of 2-AG (1 µM) (Fig. 3a-b; Relative EPSC size (norm to baseline) after 2-AG (1 µM): 0.55 ± 0.05; 2-AG + Indomethacin (5 µM): 0.50 ± 0.05, n = 5, NS by paired t-test, p = 0.19). We additionally tested the effects of 2-AG at various concentrations (10 nM, 100 nM, 1 µM, and 5 µM) after 5 min pretreatment with indomethacin (5 µM), finding that the responses were not significantly altered (Fig. 3c; EC50 for 2-AG alone (95% CI): 684 nM (216–2016); 2-AG plus indomethacin: 2315 nM (303–1770); n = 5). These experiments indicate that indomethacin is not acting as a CB1 PAM in this model.

It is possible that indomethacin exhibits either probe- or pathway-dependence. We therefore tested whether indomethacin acts as a PAM in the cAMP pathway with the synthetic agonist CP55,940 as reported by LaPrairie et al.^22^. Using the Pink Flamindo cAMP assay, we were not able to confirm a PAM-like role for indomethacin with CP55,940 (Fig. 3c; Relative AUC (Fsk = max, 100 = baseline) for CP55,940 (10 nM): 382.3 ± 36.34; CP55,940 (10 nM) + indomethacin (1 µM): 400.4 ± 30.85, NS by unpaired t-test, p = 0.78; n = 6); Fig. 3d; EC50 for CP55,940 (95%CI): 98 nM(65 nM-153 nM); CP55,940 + indomethacin: 66 nM(45 nM-97 nM) NS by overlapping 95% confidence intervals; n = 6). Summary of cAMP responses in the presence and absence of indomethacin is shown in Fig. 3e.

### The CB1 interacting protein SGIP1

#### Neuronal loss of SGIP1 impedes depolarization-induced suppression of excitation

**Figure 4:**
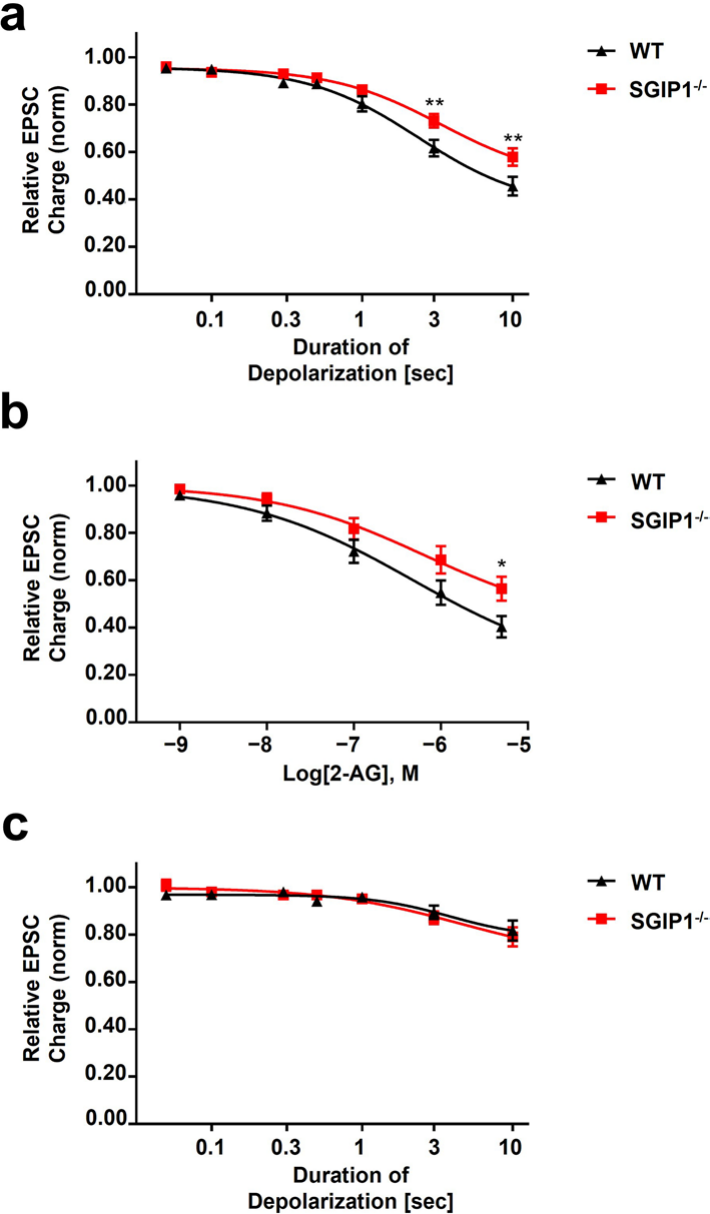
SGIP1 deletion from autaptic neurons decreases CB1-mediated synaptic plasticity and 2-AG responsiveness. Autaptic neurons were depolarized for progressively longer intervals and to induce DSE. (**a**) SGIP1^−/−^ neurons are significantly less responsive to depolarization compared to wildtype neurons. (**b**) SGIP1^−/−^ neurons are also less sensitive to 2-AG. Suppression of EPSC charge by increasing concentration of 2-AG was evaluated in autaptic neurons. (**c**) SGIP1^−/−^ neurons desensitize to the same rate and extent as WT neurons. Cells were treated overnight with the CB1 agonist WIN 55,212–2 (100 nM), washed for 20 min and DSE evaluated. Both SGIP1^−/−^ and WT neurons were almost completely desensitized by treatment with WIN55,212–2 (F(1,21) = 0.011, p = 0.918). Baseline response was normalized to 1 and DSE is plotted as fractions of 1. Data are expressed as mean ± S.E.M. (*n* = 9–24 per group). *P < 0.05, **P < 0.01, analyzed by 2-way ANOVA with Bonferroni post hoc test.

Both SGIP1 and CB1 colocalize with the presynaptic marker bassoon in neurons^31^, providing an anatomical substrate for their possible interaction. To evaluate the role of SGIP1 in neuronal CB1 function, we examined depolarization induced suppression of excitation (DSE) in neurons cultured from mice lacking SGIP1. We tested the effect of SGIP1 deletion on the DSE responses over a range of depolarizations (50 ms, 100 ms, 300 ms, 500 ms, 1 s, 3 s, 10 s). In wildtype (WT) mice, longer depolarizations yielded a successively greater inhibition of neurotransmitter release, measured as a smaller excitatory postsynaptic current (EPSC). Compared to WT, the DSE depolarization-response curve is shifted to the right in SGIP1^−/−^ neurons (F(1,36) = 4.55, p = 0.04 using 2-way ANOVA followed by Bonferroni post hoc test; ED50 WT: 2.35 s; ED50 SGIP1^−/−^: 3.34 s, Fig. 4a). Therefore, the DSE response to a given depolarization is weaker in the absence of SGIP1. This was particularly evident for longer depolarizations.

#### SGIP1 modulates 2-AG-mediated inhibition of EPSCs

2-AG activates CB1 to mediate DSE in autaptic neurons^1^. Therefore, if attenuation of DSE in SGIP1^−/−^ neurons is due to loss of its interactions with CB1, application of increasing concentrations of 2-AG to SGIP1^−/−^ neurons should result in less inhibition of EPSCs than in wildtype (WT) neurons. WT and SGIP1^−/−^ derived neurons were treated with increasing concentrations of 2-AG (1 nM-5 µM) and EPSCs were evoked every 20 s with 0 mV depolarization lasting 1 ms. In WT mice, 2-AG decreased the EPSCs in concentration-dependent manner as expected. Importantly, 2-AG was less effective in neurons lacking SGIP1 (F(1,26) = 4.28, p = 0.049 using 2-way ANOVA followed by Bonferroni post hoc test; ED50 WT: 475.5 nM, ED50 SGIP1^−/−^: 639.4 nM, Fig. 4b), reminiscent of the impaired DSE observed in SGIP1^−/−^ neurons (Fig. 4a).

#### CB1 desensitizes similarly in WT and SGIP1^−/−^ neurons

Next, we asked whether chronic application of a CB1 agonist will lead to differential desensitization of CB1 in WT compared to SGIP1^−/−^ neurons. We treated the neurons with 100 nM WIN55,212–2 (WIN) overnight, and washed away WIN for at least 20 min before measuring DSE responses. The SGIP1^−/−^ neurons, similar to the WT neurons were almost completely desensitized by this treatment (F(1,21) = 0.011, p = 0.92 using 2-way ANOVA, Fig. 4c).

The results from electrophysiological experiments suggest that SGIP1 is needed to maintain the full efficacy of eCB signaling at CB1 but is dispensable for CB1 desensitization.

## Discussion

Over the course of more than twenty years we have made use of the autaptic hippocampal model to study various aspects of cannabinoid signaling system and its pharmacology in a tractable neuronal setting. Initially the model helped identify the molecular machinery of cannabinoid signaling and plasticity, this at a time when the very identity of the endogenous messenger remained uncertain. Once characterized, the autaptics served as an attractive platform for pharmacological studies, testing interaction not only with CB1 receptors in a native neuronal environment but also the enzymatic and transport machinery that synthesize, delivers, and metabolize 2-AG. Over the years we have noted findings that, while interesting, were unlikely to find their way into a manuscript as standalone datasets. The findings we report here fall into two broad categories: agents/proteins that might act on CB1 receptors (indomethacin, 1-AG, and SGIP1) and a compound that has been proposed to transport 2-AG. In each case we find that these act in unexpected ways. Because the results reflect very different topics, we will address each separately below.

### Cannabinoid transport

One of the continuing mysteries of cannabinoid signaling has to do with how endogenous cannabinoids make their way from the interior of postsynaptic dendrites to the presynaptic terminal to activate the transmembrane CB1 receptors. Synthesis of 2-AG, the endocannabinoid that is clearly implicated in autaptic cannabinoid signaling is considered to occur via the activity of diacylglycerol lipases^3^. What happens after its synthesis is largely a mystery, but chaperones and transporters have been proposed at various instances. The rationale is that as lipophilic messengers, endocannabinoids would be reluctant to traverse the membrane into a hydrophilic environment and that a transporter or chaperone might facilitate or even guide endocannabinoids across the membrane and to presynaptic CB1 receptors.

FABPs are proposed to act as intracellular chaperones for anandamide^13^ but are also implicated in several important physiological functions, some of which may involve 2-AG-mediated cannabinoid signaling (reviewed in^14^). In particular FABP5 deletion has been shown to interfere with 2-AG-mediated depolarization induced suppression of inhibition (DSI) in striatal medium spiny neurons without impacting CB1 receptor function^16^. From this, and similar results in excitatory neurons of the dorsal raphe nucleus^17^, it was proposed that FABP5 may generally facilitate the transfer of 2-AG across the synaptic cleft. This is an exciting proposition and it predicts that in autaptics an FABP5 blocker would inhibit 2-AG-mediated DSE.

Autaptic hippocampal neurons seem well-suited as a platform to test such compound since they express all the machinery necessary for synthesis, retrograde mobilization, and presynaptic metabolism of 2-AG. But SBFI26 did not affect autaptic cannabinoid responses. It is possible that FABP5 plays a regionally specific role, i.e. that retrograde cannabinoid signaling in pyramidal hippocampal neurons is somehow distinct from that in the striatum and the dorsal raphe nucleus. It also is possible that some other FABP serves a comparable role in the hippocampus. Deletion of FABP5, as reported in Fauzan et al.^16^, may also cause different effects than acute pharmacological blockade.

### Indomethacin does not act as a PAM in this model

The autaptic model serves as an attractive model for studies of CB1 receptor allosteric modulation^8,9,23,39^. The report by Laprairie et al.^22^ that indomethacin acts as a positive allosteric modulator (PAM) was provocative and exciting and would be of particular interest in a neuronal context given the broad use of indomethacin for analgesia. Here we show that indomethacin does not enhance 2-AG mediated signaling in autaptic neurons. Our results argue against a CB1 PAM role for the form of cannabinoid-mediated neuronal plasticity assessed here, but there are important limitations to the interpretation of our findings. Indomethacin may exhibit either probe- and/or pathway-dependence, something seen for other allosteric modulators (reviewed in^40^). Regarding the former, Laprairie et al. tested the interaction of indomethacin with anandamide whereas the autaptic system makes use of 2-AG. In consideration of this we tested indomethacin interaction with the CB1 agonist CP55940 in a cAMP assay, mimicking the ligand and signaling pathway. We therefore did not see an effect for indomethacin even under conditions that mimicked the published findings for indomethacin^22^.

### 1-AG is a weak agonist for endogenous CB1 receptors in autaptic neurons

Though 2-AG is now an accepted endogenous messenger acting on CB1 receptors in the CNS, there are some peculiarities about 2-AG that remain incompletely addressed. One question has to do with disposition of the isomer 1-AG. Several studies have shown evidence that 1-AG is active. In addition to the reports from Stella et al., Sugiura et al.^19,41^ offered evidence for functional signaling of 1-AG, though it emphasized the primacy of 2-AG. A separate study by Savinainen et al.^42^ examined 1-AG-stimulated GTPγS binding and found 2-AG to be more potent and efficacious than 1-AG. Despite this evidence that 1-AG is capable of functionally activating cannabinoid receptors, 1-AG is described in several publications as an inactive metabolite^43,44^.

This may be in part because van der Stelt^45^ reported that 1-AG does not bind the CB_1_ receptor as measured by displacement of tritiated CP55,940 from rat forebrain membranes, a surprising finding given the aforementioned functional studies. One possibility that may explain biological activity of 1-AG is that, like 2-AG^46^, 1-AG activates TRPV1 receptors^47^. Additionally, another significant issue is whether 1-AG is even present under physiological conditions. Stella et al.^19^ concluded that the 1-AG in their samples could be accounted for by post-extraction isomerization. This and several subsequent studies^48,49^ assumed that 1-AG could safely be folded into the totals for 2-AG and by implication that 1-AG, regardless of activity, is simply not present in sufficient quantity to play a significant physiological role.

In the autaptic model, 1-AG signaled poorly relative to 2-AG and the inhibition seen is comparable to what might be expected from the 10% contamination of the 1-AG solution by 2-AG. We consider this a likely explanation for our findings; in which case it is probable that 1-AG signals even less in this system than would be inferred from our results. It is possible that the autaptic preparation has a limited number of CB1 receptors and/or weak receptor coupling that may exaggerate the difference between 1-AG and 2-AG if 1-AG has a lower intrinsic efficacy than 2-AG. 1-AG may also act via other pathways but our results argue against a substantial role for 1-AG in regulating neuronal retrograde inhibition, a major functional role for 2-AG in the CNS^50^.

### SGIP1 affects the acute phase of CB1 signaling in autaptic neurons but not its desensitization

As noted above, previous studies have implicated SGIP1 in cannabinoid receptor recycling. Deletion of SGIP1 in mice affects their behavior and nociception which highlights the regulatory role of SGIP1 in cannabinoid signaling^55^. The prediction based on prior findings is that SGIP1 would enhance CB1 signaling by increasing the ‘dwell time’ in the plasma membrane. In principle our finding that WT neurons with functional SGIP1 exhibit stronger DSE than neurons lacking SGIP1 is consistent with this hypothesis. However, the same hypothesis also predicts altered desensitization of the receptor, something that we did not observe. One possible explanation of the lack of the effect of SGIP1 deletion could be that SGIP1 may not be involved in desensitization and rather, by impeding CB1 internalization, may affect cannabinoid signaling on different levels (e.g., constitutive activity of CB1 or signaling from endosomes).

## Summary

For more than 20 years and in more than 25 publications, the autaptic hippocampal model has proven to be valuable to characterize CB1- and 2-AG-dependent cannabinoid signaling in a neuronal setting. Here we have reported several ‘orphan’ findings deriving from studies of cannabinoid signaling using autaptic hippocampal neurons. These findings, though interesting, might not have found their way into a publication on their own. It is our hope that these findings will prove beneficial to other laboratories and elicit fruitful discussions regarding their relevance and significance. The reader should however keep in mind that the autaptic preparation is a simplified model and despite possessing all the components of natively expressed endocannabinoid system there still might be some limitations to data obtained from such cells (as discussed in the relevant subchapters).

## Data Availability

The datasets used and/or analyzed during the current study are available from the corresponding author on reasonable request.
